# Biclonal myelodysplastic syndrome involving six chromosomes and monoallelic loss of RB1 - A rare case

**DOI:** 10.1186/1755-8166-4-16

**Published:** 2011-08-18

**Authors:** Walid Al-Achkar, Abdulsamad Wafa, Elisabeth Klein, Abdulmunim Aljapawe

**Affiliations:** 1 Molecular Biology and Biotechnology Department, Human Genetics Division, Atomic Energy Commission, Damascus, Syria; 2 Jena University Hospital, Institute of Human Genetics, Jena, Germany; 3 Molecular Biology and Biotechnology Department, Mammalians Biology Division, Atomic Energy Commission, Damascus, Syria

## Abstract

**Background:**

Myelodysplastic syndrome (MDS) represents a group of clonal hematological disorders characterized by progressive cytopenia, and reflects to defects in erythroid, myeloid and megakaryocytic maturation. MDS is more frequently observed in older aged patients with cytogenetic abnormalities like monosomy of chromosome(s) 5 and/or 7. In 50% of de novo MDS cases, chromosomal aberrations are found and rearrangements involving the retinoblastoma (*RB1*) gene in 13q14 are found.

**Results:**

Here, we are presenting a case report of a rare biclonal MDS with a karyotype of 45, XY,-4, der(6)t(4;6)(p15.1;p21.3), der(8)t(4;8)(q31.2;q22), t(13;16)(q21.3;p11.2)[11]/45, XY, der(7)t(7;13)(p22.2~22.3;q21.3),-13 [9]. The patient was diagnosed according to WHO classification as refractory anemia with excess of blasts (RAEB-II).

Immunophenotyping was positive for CD11b, CD11c, CD10, CD13, CD15, CD16 and CD33.

**Conclusion:**

We report, a novel and cytogenetically rare case of a biclonal MDS with complex chromosomal aberrations and deletion of *RB1*-gene in both clones. These findings are associated with a poor prognosis as the patient died 3 months after diagnosis.

## Background

Myelodysplastic syndrome (MDS) refers to a group of clonal acquired diseases characterized by trilineage defects in erythrocytic, granulocytic, and megakaryocytic lineages of hematopoiesis. Although considerable as a clonal malignancy of its own, MDS is sometimes classified as a premalignant condition, which progresses to acute myeloid leukemia (AML) regularly [1]. Overall, MDS affects approximately 1 in 500 persons over 60 years of age, making it the most common hematologic malignancy in this age group [1].

Cytogenetic abnormalities are found in ~50% of the patients with de novo MDS and the most commonly involved chromosomal changes observed are monosomy 5 and/or 7, trisomy 8 and/or partial deletion in 5q, 7q, 9q and 20q [2,3]. Unbalanced translocations are also frequently found, and they are usually detected as a part of complex karyotypes, associated with loss of chromosomal material, and related to disease progression [4].

Deletions or translocations involving chromosomal band 13q14, the locus of the retinoblastoma (*RB1*) gene, are observed in a variety of hematological malignancies including myelofibrosis (MF), MDS, AML, chronic myelogenous leukemia (CML) and chronic lymphocytic leukemia (CLL) [4]. Recently, it has been shown that deletions of 13q14 are detected at a high frequency (more than 40%) in cases of CLL and multiple myeloma (MM) by fluorescence *in situ *hybridization (FISH) analyses [4].

We are presenting a new case of a biclonal MDS case with yet unreported translocation events involving six different chromosomes and a monoallelic loss of *RB1 *in both clones. In this case, multicolor banding (MCB) technique was found very useful for characterizing the breakpoints involved in the chromosomal rearrangements in this case.

## Case report

In June 2009, a 60 year old male patient was referred with anemia, thrombocytopenia, loss of weight and fever. His white blood cell count was 7 × 10^9^/l, with 64.5% neutrophils, 24.6% lymphocytes, 4.2% monocytes, 1% eosinophiles, 1% basophils and 4.6% blast. Bone marrow was hypercellular with 19% blast cells. Dysplastic changes in bone marrow included cytoplasmic hypogranulation of neutrophils, erythroblasts and micromegakaryocytes. The red blood cell count was 3.38 × 10^6^/cmm with 8.3 g/dl hemoglobin level along with platelet count of 49 × 10^9^/l, and LDH value of 571 U/l. Physical examination of the patient showed splenomegaly. The patient was treated with Zyloric (300 mg as a daily dose) and Hydroxyurea (500 mg as a daily dose) for 1 month and later continued on Hydroxyurea (500 mg as a daily dose) for 3 month. The patient died 3 months after diagnosis.

**Figure 1:**
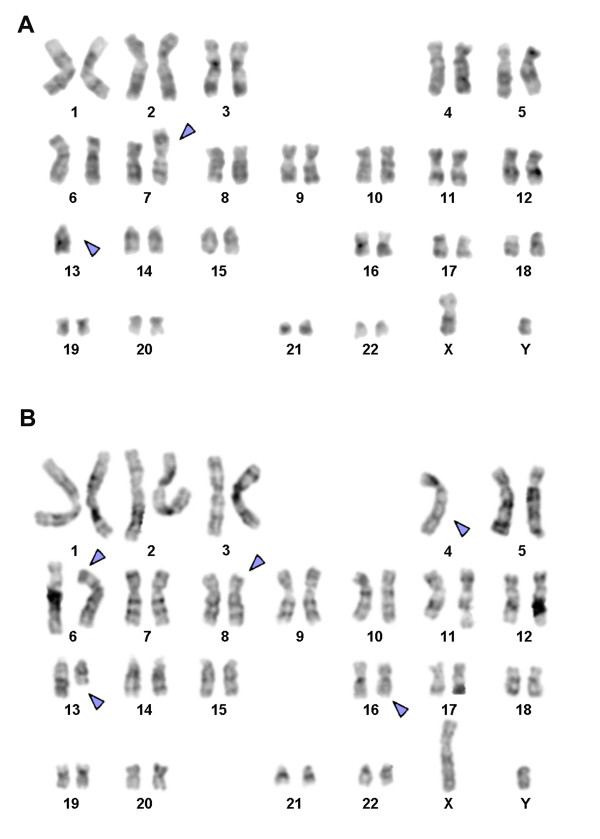
**GTG-banding revealed a biclonal condition in the present case**. All derivative chromosomes are marked by an arrow head. (A) A translocation between chromosomes 7, 13 and loss of one chromosome 13 was observed. (B) A complex karyotype involving the chromosomes 4, 6, 8, 13 and 16.

**Figure 2:**
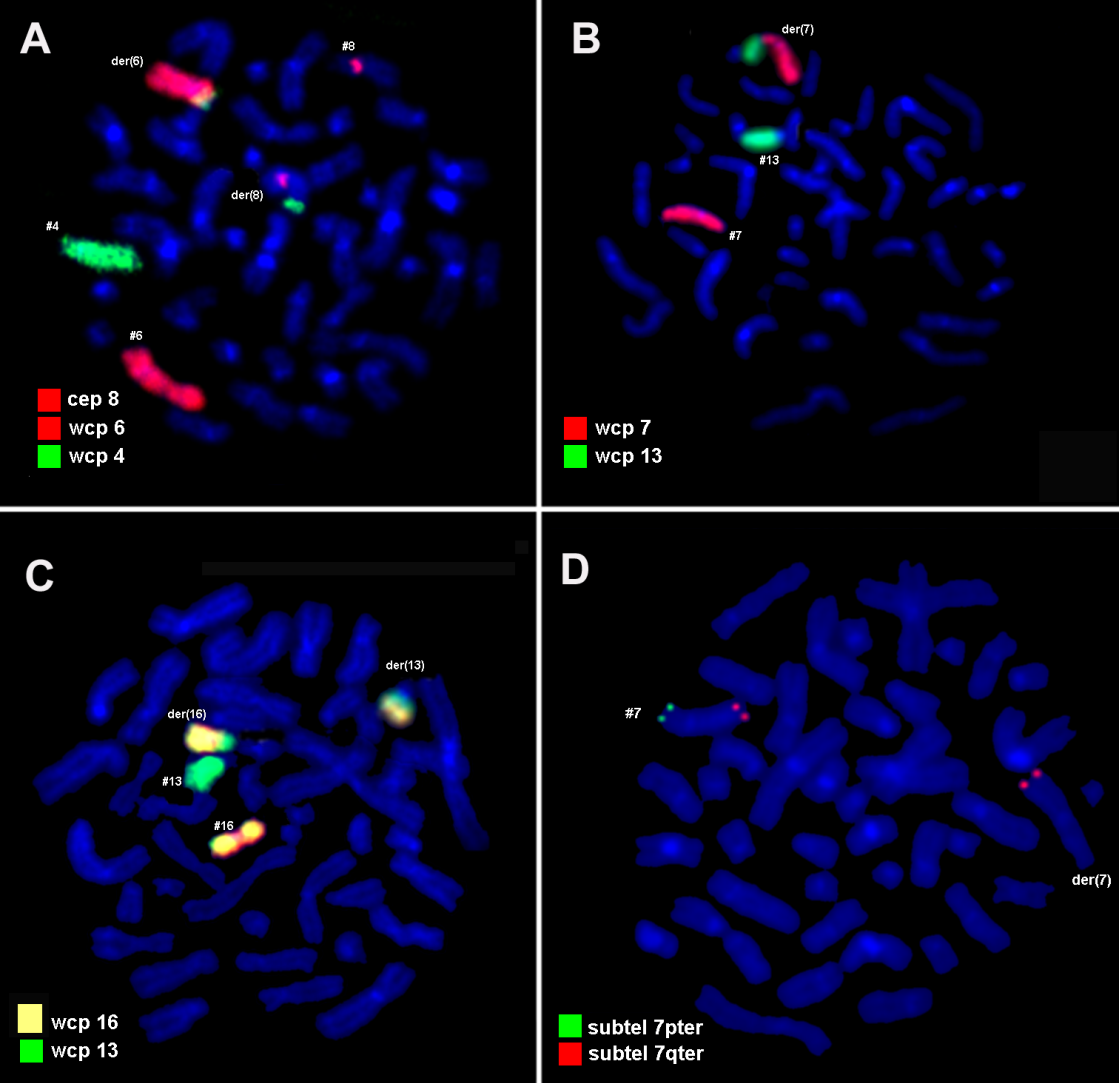
**Karyotype and chromosomal aberrations were confirmed using molecular cytogenetic approach**. (A) The translocation among chromosomes 4, 6 and 8 were identified using WCP for chromosomes 4 and 6 (MetaSystems, Altlussheim, Germany) mixed with CEP 8 (Abbott Molecular/Vysis, USA). (B) The translocation between chromosomes 7 and 13 was identified using WCP for chromosomes 7 and 13 (MetaSystems, Altlussheim, Germany). (C) The translocation between chromosomes 13 and 16 was identified using WCP for chromosomes 13 and 16 (MetaSystems, Altlussheim, Germany). (D) The subtelomeric deletion of chromosome 7 was identified using subtelomeric 7pter and subtelomeric 7qter (Abbott Molecular/Vysis, USA). Abbreviations: #, chromosome; der, derivative chromosome.

**Figure 3:**
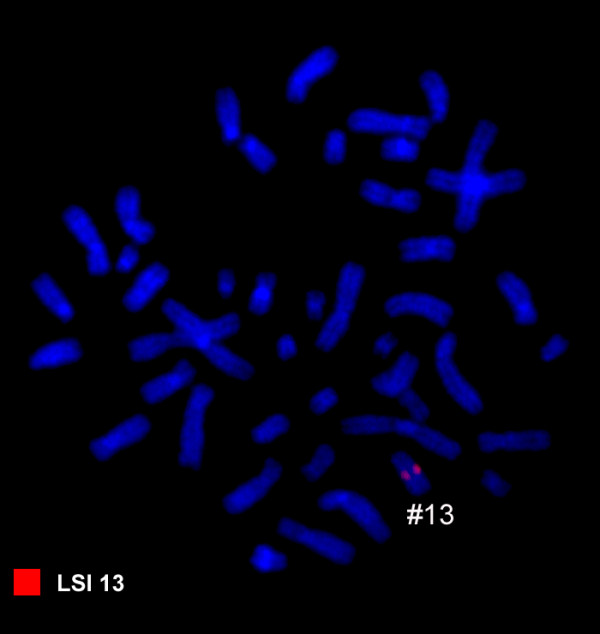
**Fluorescence *in situ *hybridization (FISH) using probe for RB1 gene on metaphase spread showed one RB1 signal on normal chromosome 13 in the second clone**. Abbreviations: #, chromosome.

**Figure 4:**
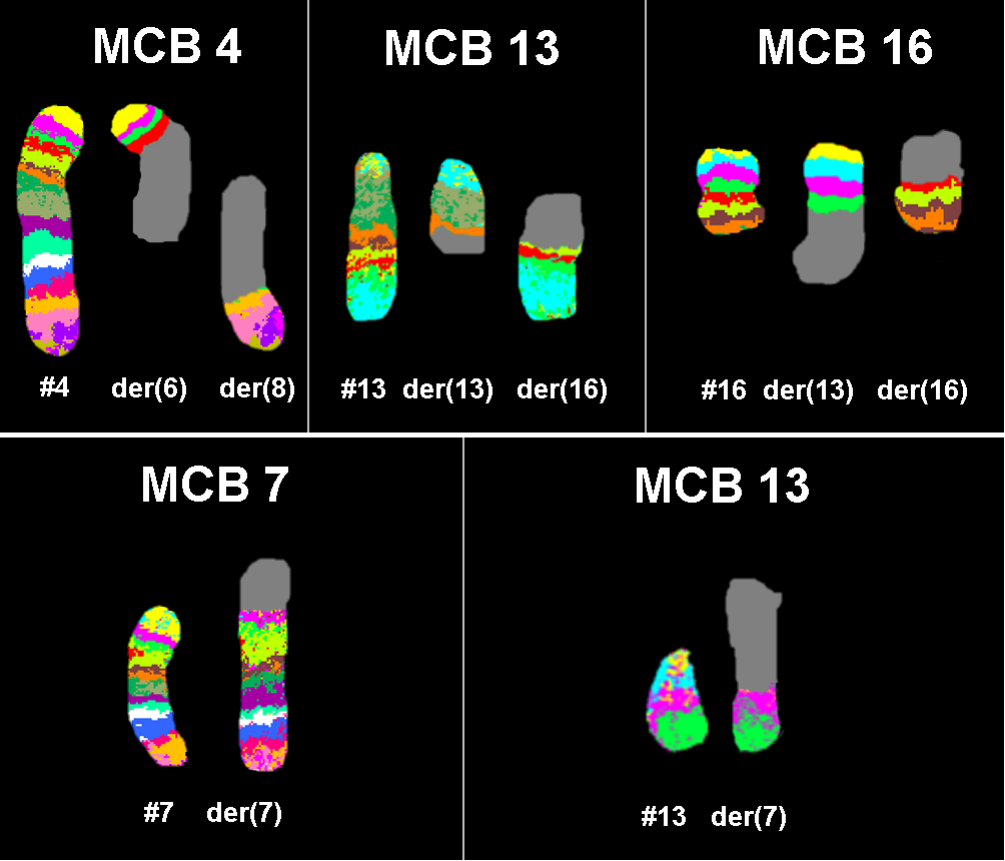
**Multicolor banding (MCB) was applied to determine which chromosomes were involved in the chromosomal aberrations**. Each image shows the results of MCB analysis using probe sets for chromosomes 4, 7, 13 and 16. The normal chromosomes are shown in the left side of each image and the derivative of the four chromosomes on the right side of normal chromosomes. The MCB-probes unstained regions on the derivative chromosomes are shown in gray. #, chromosome; der, derivative chromosome.

Karyotyping was done after the initiation of the treatment which showed a mosaic and biclonal karyotype with 45, XY, -4, der(6)t(4;6)(?;?), der(8)t(4;8)(?;?), t(13;16)(?;?)/45, XY, der(7)t(7;13)(?;?),-13 (Figure 1), which was further studied by molecular cytogenetics (Figure 2, 3, 4). Dual-color-FISH using WCP and CEP probes specific for chromosomes 4, 6, 7, 8, 13 and 16 confirmed the translocation seen in GTG-banding. Application of subtelomeric probes for 7pter and 7qter revealed two signals of subtelomeric 7qter on both homologous chromosomes 7 and one signal of subtelomeric 7pter on intact chromosome 7. Thus, subtelomeric region 7pter was deleted on the derivative chromosome 7 (Figure 2). Applying an RB1-specific probe showed one signal only on the normal chromosome 13 in both clones. The analysis using MCB probes specific for individual chromosome involved in translocation, determined the breakpoint location and the final karyotype was found to be with 45, XY, -4, der(6)t(4;6)(p15.1;p21.3), der(8)t(4;8)(q31.2;q22), t(13;16)(q21.3;p11.2)[11]/45, XY, der(7)t(7;13)(p22.2~22.3;q21.3),-13 [9].

Immunophenotyping of peripheral blood characterized the neutrophiles which showed abnormal side scatter pattern, as well as abnormal intensity staining patterns for CD11b(63%), CD11c(59.3%), CD10(17.4%), CD13(44%), CD15(59.3%), CD16(46%) and CD33(20.4%). The majority of monocytes were HLADr+ (4.2%). Lymphocyte subsets percentages were low. The patient was diagnosed as having common MDS, refractory anemia with excess of blasts (RAEB) in the French-American-British (FAB) classification, or RAEB-II in the World Health Organization (WHO) classification [5].

## Discussion

We described a biclonal MDS case with cytogenetic rearrangements involving six different chromosomes together with a monoallelic loss of the RB1 gene in both clones.

The Cancer Genome Anatomy Project databases (http://cgap.nci.nih.gov/Chromosomes/AbnCytSearchForm) and atlas of genetics and cytogenetics in oncology and hematology (http://atlasgeneticsoncology.org/) showed not a single case of MDS with a der(6)t(4;6)(p15.1;p21.3), a der(7)t(7;13)(p22.2~22.3;q21.3), a der(8)t(4;8)(q31.2;q22), or a t(13;16)(q21.3;p11.2). Contrarily the involvement of *RB1 *gene in MDS is known and appears to be a rare event [4]. The RB1 protein acts as a cell cycle regulator which blocks the transition of normal cells from G0/G1 into S phase of the cycle. Mice with homozygous disruption of the RB1 alleles resulted in an overall normal development but had lethal anemia, suggesting a critical role of the RB1 gene in erythropoiesis [4]. In the present case, anemia and thrombocytopenia were predominantly observed during the clinical course, while white blood cells count was relatively preserved. This impaired erythropoiesis might be related to monoallelic loss of the RB1 gene [4].

Besides imbalances of chromosomes 13, the observed rearrangements lead to a partial monosomy 4p15.1 to 4q31.2 in the slightly larger of both clones. In accordance with the international prognostic scoring system (IPSS) classification of chromosomes 4 and 13 loss in a RAEB-II stage patient supports an intermediate-2 prognosis group and/or poor prognosis group [6,7].

The decreased heterogeneous expression of those antigens was consistent with myelodysplastic disease in transition.

Concerning the additional observed acquired imbalances, up to present somehow similar minor terminal deletions of 6p21.3 in malignant hematological disorders was observed, and an involvement of the breakpoint 8q22 in an oncogene induced solid tumor [8,9]. Deletions of 7p confer an inferior outcome in children with ALL, regardless of the presence of other poor prognostic features. Monosomy 7 is also associated with inferior event-free survival (EFS) in children with ALL [10]. 13q21.3 region was involved in CLL cases and 16p11.2 region was found in classical Hodgkin lymphoma but to our knowledge our breakpoints have not been reported in MDS, yet [11,12].

According to a recent study, the LDH level was nearly as powerful as a prognostic parameter as karyotyping and an elevated LDH was associated with poor prognosis in MDS [13]. The LDH level for the presented patient was 571 U/l, which compared to the normal value (up to 480 U/l) is enhanced. Thus, also the LDH level as well as cytogenetics supported an adverse prognosis, which unfortunately was confirmed by the clinical outcome.

In conclusion, here we reported a novel translocation involving six chromosomes distributed in two clones and monoallelic loss of RB1 in both clones. Our finding and according to WHO classification, IPSS and LDH is considered to a poor prognostic factor in MDS patients, as no response was observed after the application of chemotherapy.

## Materials and methods

### Chromosome analysis

Chromosome analysis using GTG-banding was performed according to standard procedures [14]. Twenty metaphases derived from unstimulated bone marrow of the patient were analyzed. Karyotypes were described according to the International System for Human Cytogenetic Nomenclature [15].

### Molecular cytogenetics

Fluorescence *in situ *hybridization (FISH) using whole chromosome painting (WCP) probe for chromosomes 4, 6, 7, 13 and 16 (MetaSystems/Germany) and subtelomeric probes for 7pter and 7qter (Abbott Molecular/Vysis, USA) were applied according to manufacturer's instructions together with a chromosome enumeration probe (CEP) for chromosome 8 and specific probe for RB1 (LSI 13 (RB1) Abbott Molecular/Vysis, USA) [14]. Multicolor banding probe (MCB) sets based on microdissection derived region-specific libraries for chromosome 4, 7, 13 and 16 were applied as previously described [16]. Twenty metaphase spreads were analyzed, each using a fluorescence microscope (AxioImager.Z1 mot, Zeiss) equipped with appropriate filter sets. Image capturing and processing were carried out using an Isis mFISH imaging system (MetaSystems, Altlussheim, Germany).

### Immunophenotyping

Immunophenotyping of leukemic blasts was done using general panel of fluorescent antibodies against the following antigens typical for different cell lineages and cell types: CD1a, CD2, CD3, CD4, CD5, CD8, CD10, CD11b, CD11c, CD13, CD14, CD15, CD16, CD19, CD20, CD22, CD23, CD32, CD33, CD34, CD38, CD41a, CD45, CD56, CD57, CD64, CD103, CD117, CD123, CD209, CD235a and CD243; In addition to antibodies to Kappa and Lambda light Chains, sIgD, sIgM, and HLADr. All antibodies were product from BD Biosciences. Four-color immunophenotyping on peripheral blood specimen was performed. Samples stained and analyzed on a BD FACSCalibur™ flow cytometer according to BD Biosciences manuals and products insert sheets. Autofluorescence, viability, and isotype controls were included. Flow cytometric data acquisition and analysis conducted by BD Cellquest™ Pro software.

## Competing interests

The authors declare that they have no competing interests.

## Authors' contributions

AW performed the cytogenetic studies in the present case and collected the data relative to this case report; WA supervised the cytogenetic analysis; AW, EK did the molecular cytogenetic analysis and interpretation; AA did the flow cytometry analysis and AW drafted the paper and all authors contributed to the finalizing of the manuscript. All authors read and approved the final manuscript.

## Consent

Written informed consent was obtained from the patient for publication of this case report and accompanying images. A copy of the written consent is available for review by the Editor-in-Chief of this journal.
